# Self-sustaining IL-8 loops drive a prothrombotic neutrophil phenotype in severe COVID-19

**DOI:** 10.1172/jci.insight.150862

**Published:** 2021-09-22

**Authors:** Rainer Kaiser, Alexander Leunig, Kami Pekayvaz, Oliver Popp, Markus Joppich, Vivien Polewka, Raphael Escaig, Afra Anjum, Marie-Louise Hoffknecht, Christoph Gold, Sophia Brambs, Anouk Engel, Sven Stockhausen, Viktoria Knottenberg, Anna Titova, Mohamed Haji, Clemens Scherer, Maximilian Muenchhoff, Johannes C. Hellmuth, Kathrin Saar, Benjamin Schubert, Anne Hilgendorff, Christian Schulz, Stefan Kääb, Ralf Zimmer, Norbert Hübner, Steffen Massberg, Philipp Mertins, Leo Nicolai, Konstantin Stark

**Affiliations:** 1 Department of Medicine I, University Hospital, Ludwig-Maximilians University Munich, Germany.; 2 German Centre for Cardiovascular Research (DZHK), partner site Munich Heart Alliance, Munich, Germany.; 3 COVID-19 Registry of the LMU Munich (CORKUM), University Hospital, Ludwig-Maximilians University Munich, Munich, Germany.; 4 Max Delbrück Center for Molecular Medicine (MDC) in the Helmholtz Association, Berlin, Germany.; 5 DZHK, partner site Berlin, Berlin, Germany.; 6 Department of Informatics, Ludwig-Maximilians University Munich, Munich, Germany.; 7 Max von Pettenkofer Institute and GeneCenter, Virology, Faculty of Medicine, Ludwig-Maximilians University, Munich, Germany.; 8 German Center for Infection Research, Partner Site Munich, Munich, Germany.; 9 Medical Clinic and Polyclinic III, University Hospital, Ludwig-Maximilians University Munich, Munich, Germany.; 10 Institute of Computational Biology, Helmholtz Zentrum München (German Research Center for Environmental Health), Neuherberg, Germany.; 11 Department of Mathematics, Technical University of Munich, Garching, Germany.; 12 The COMBAT C19IR study group is detailed in the Acknowledgments.; 13 Institute for Lung Biology and Disease and Comprehensive Pneumology Center with the CPC-M bioArchive, Helmholtz Center Munich, Member of the German Center for Lung Research, Munich, Germany.; 14 Center for Comprehensive Developmental Care at the interdisciplinary Social Pediatric Center, Haunersches Children’s Hospital, University Hospital Ludwig-Maximilian University, Munich, Germany.; 15 Charité-Universitätsmedizin Berlin, Germany.

**Keywords:** COVID-19, Vascular Biology, Cytokines, Neutrophils, Proteomics

## Abstract

Neutrophils provide a critical line of defense in immune responses to various pathogens, inflicting self-damage upon transition to a hyperactivated, procoagulant state. Recent work has highlighted proinflammatory neutrophil phenotypes contributing to lung injury and acute respiratory distress syndrome (ARDS) in patients with coronavirus disease 2019 (COVID-19). Here, we use state-of-the art mass spectrometry–based proteomics and transcriptomic and correlative analyses as well as functional in vitro and in vivo studies to dissect how neutrophils contribute to the progression to severe COVID-19. We identify a reinforcing loop of both systemic and neutrophil intrinsic IL-8 (CXCL8/IL-8) dysregulation, which initiates and perpetuates neutrophil-driven immunopathology. This positive feedback loop of systemic and neutrophil autocrine IL-8 production leads to an activated, prothrombotic neutrophil phenotype characterized by degranulation and neutrophil extracellular trap (NET) formation. In severe COVID-19, neutrophils directly initiate the coagulation and complement cascade, highlighting a link to the immunothrombotic state observed in these patients. Targeting the IL-8–CXCR-1/-2 axis interferes with this vicious cycle and attenuates neutrophil activation, degranulation, NETosis, and IL-8 release. Finally, we show that blocking IL-8–like signaling reduces severe acute respiratory distress syndrome of coronavirus 2 (SARS-CoV-2) spike protein–induced, human ACE2–dependent pulmonary microthrombosis in mice. In summary, our data provide comprehensive insights into the activation mechanisms of neutrophils in COVID-19 and uncover a self-sustaining neutrophil–IL-8 axis as a promising therapeutic target in severe SARS-CoV-2 infection.

## Graphical abstract

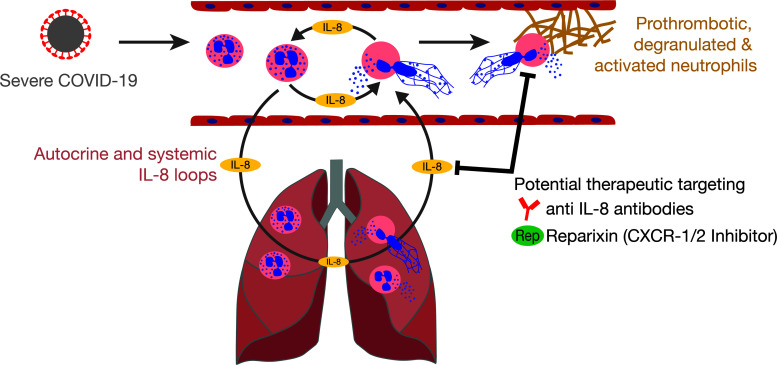

## Introduction

Neutrophils are first responders to invading pathogens, with either their absence or dysfunction resulting in an immunocompromised state (1). In viral infections, neutrophils are quickly recruited, ameliorate local tissue damage, and limit systemic viral spread by contributing to both clearance of infected tissue and production of antiviral cytokines (2, 3). However, their antimicrobial activity also causes harm. In particular, the excessive release of neutrophil extracellular traps (NETs) and cytotoxic granule contents contributes to self-inflicted damage to both local and remote organs (4–6). This process of immunopathology is a hallmark of severe coronavirus disease 2019 (COVID-19) and contributes to respiratory failure, progressive immune dysregulation and — ultimately — death (7–10). Therefore, limiting immunopathology might be a promising therapeutic strategy to improve outcomes in COVID-19. This is supported by the efficacy of corticosteroid treatment in pneumonic COVID-19 (11). Consequently, it is crucial to increase our understanding of immune dysregulation in critical cases of severe acute respiratory distress syndrome of coronavirus 2 (SARS-CoV-2) infection.

We and others have shown that neutrophil hyperactivation and NET formation contribute to the occurrence of immunothrombosis in COVID-19 and correlate with disease severity (12–15). Transcriptomics have revealed striking changes in the neutrophil compartment, including mobilization of bone marrow–derived immature neutrophils with a unique proinflammatory signature (9, 16). Nevertheless, neutrophil protein composition and content, granule release, and recruitment mechanism data across COVID-19 disease states are scarce.

Here, we use state-of-the-art mass spectrometry–based proteomics, transcriptomic, and correlative analyses of clinical data combined with functional in vitro and in vivo studies to characterize neutrophils in COVID-19. In severe disease, we demonstrate a degranulated, prothrombotic neutrophil phenotype in peripheral blood characterized by upregulation of pathways associated with the response to and production of IL-8. Neutrophils recruited to the lung express IL-8, which in turn activates and enhances IL-8 release of peripheral neutrophils. In line with this finding, we identify increased plasma IL-8 levels in a cohort of patients with COVID-19 with severe disease but no increase in patients with mild-to-moderate disease. Blocking this pathway attenuates NET formation, degranulation, and neutrophil activation. Finally, targeting the IL-8 receptors CXCR-1/-2 in a murine model of SARS-CoV-2 spike protein–induced lung injury ameliorates neutrophil-driven pulmonary microthrombosis. In summary, our data suggest a systemic and autocrine IL-8–CXCR-1/-2 axis at the center of neutrophil-driven immunopathology in severe COVID-19.

## Results

### Neutrophil proteomics reveal strong IFN responses and enhanced IL-8 signaling in COVID-19.

Because neutrophils are a main cellular factor contributing to disease progression in COVID-19, we set out to investigate their functional status at the protein level in patient samples. To this end, we recruited patients with COVID-19 with intermediate or severe disease and compared them with patients hospitalized for non–COVID-19 viral pneumonias (Pneu) and with healthy controls (Ctrl). Patients with COVID-19 requiring normal ward inpatient hospital care were grouped into the intermediate cohort (Int), patients requiring additional invasive ventilation or intensive care treatment were included in the severe cohort (Sev). We profiled the proteome of peripheral blood neutrophils using tandem mass tag mass spectrometry (Supplemental Figure 1, A and B; supplemental material available online with this article; https://doi.org/10.1172/jci.insight.150862DS1). A total of 5087 neutrophil proteins were identified. Our comprehensive neutrophil protein atlas is available online at https://neuprocov.mdc-berlin.de and includes a graphical interface to interrogate neutrophil protein dynamics in response to SARS-CoV-2 and other viral pneumonias compared with healthy controls.

**Figure 1:**
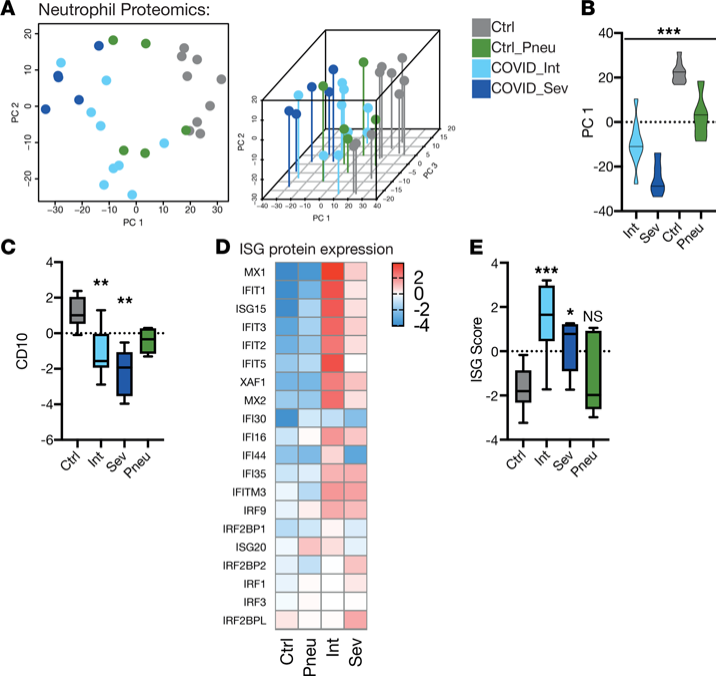
Neutrophil proteomics reveal immature neutrophils with a strong IFN response in COVID-19. (**A**) Principal component analysis of neutrophils of patients, by group. Proteins with adjusted P value of less than 0.1 were included for this PCA. (**B**) Positions of patient neutrophil samples on principal component axis 1 (PC 1). One-way ANOVA. (**C**) Box plot of CD10 abundance (log-scaled median-MAD normalized abundance) of neutrophils by group. Abundance is normalized with median-MAD method. Significance is adjusted *P* values. (**D**) Heat map of mean IFN-stimulated genes (ISG) protein abundance on neutrophils by group. (**E**) Box plot of ISG score calculated from log-scaled abundance values (see methods) on neutrophils by group. One-way ANOVA with post hoc Holm-Sidak’s multiple comparisons test to control. (**A–E**), *n* = 9 healthy control, *n* = 5 pneumonic control, *n* = 5 severe COVID-19 and *n* = 9 intermediate patients with COVID-19. *P < 0.05, **P < 0.01, ***P < 0.001.

3D principal component analysis (PCA) of all proteins and differentially regulated proteins separated neutrophils of patients with COVID-19 from both healthy control patients and patients with non–COVID-19 pneumonia (Figure 1A and Supplemental Figure 1D). In addition, we observed a trend toward differential clustering according to disease severity in COVID-19 (Figure 1, A and B, and Supplemental Figure 1, C–E). Sorted cells also included immature neutrophils characterized by low expression of membrane metalloendopeptidase (MME)/CD10, a neutrophil subset that has been described to be especially prominent in COVID-19 (Figure 1C) (17).

Recently, several landmark studies have revealed the role of IFN-induced transcriptional changes in gene expression and the immune response to SARS-CoV-2 infection (18–21). We confirmed this finding at the protein level: Neutrophils from patients with COVID-19 were enriched for IFN stimulated gene (ISG) products, with both intermediate and severe cases, compared with control patients without COVID-19, exhibiting elevated levels of canonical ISG products, such as IFN-induced protein with tetratricopeptide repeats 1 (IFIT1), IFN-induced GTP-binding protein MX1 and IFN-induced transmembrane protein 3 (IFITM3, Figure 1D) (22), and, consequently, a marked increase in computed ISG scores (Figure 1E). We and others have hypothesized that an early and strong upregulation of ISGs is a hallmark of protective immune response to SARS-CoV-2, thereby preventing systemic inflammatory spillover associated with severe COVID-19 cases (16, 23). In line with this concept, neutrophils from mild-to-moderate cases exhibited stronger IFN responses compared with neutrophils from patients requiring intensive care treatment (Figure 1E).

**Figure 2:**
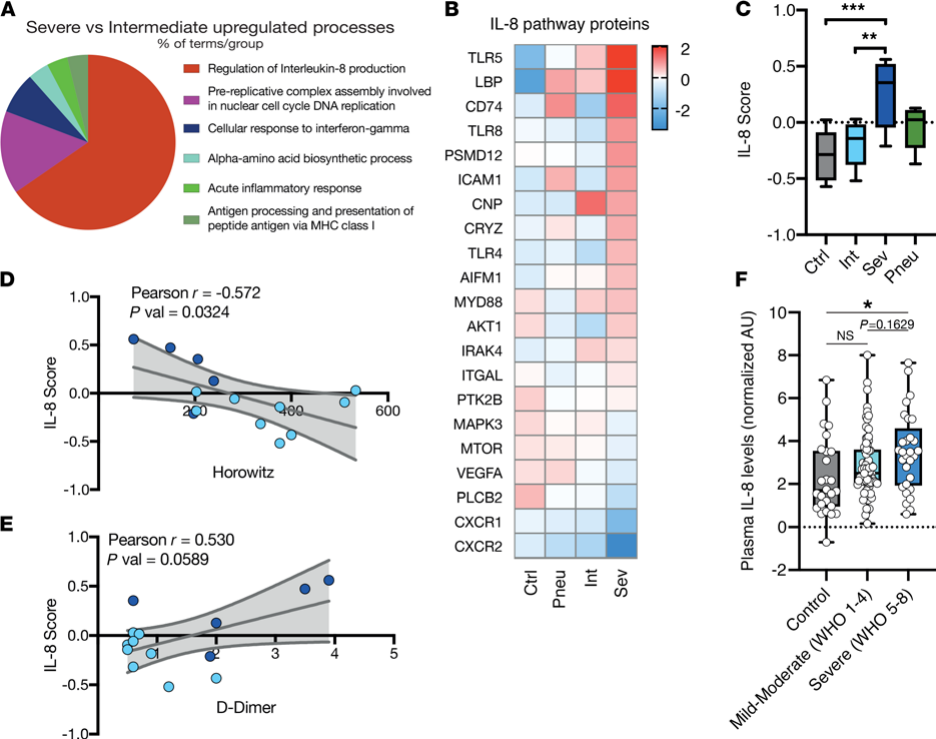
Severe COVID-19 neutrophils upregulate proteins implicated in IL-8 signaling. (**A**) ClueGo upregulated neutrophil proteome pathway grouping of severe COVID-19 compared with intermediate COVID-19. (**B**) Heatmap of mean IL-8 pathway protein abundance on neutrophils by group. (**C**) Box plot of IL-8 score calculated from log-scaled abundance values (see methods) by group. One-way ANOVA with post hoc Tukey’s multiple comparisons test between all groups. (**A–C**) *n* = 9 healthy control, *n* = 5 pneumonic control, *n* = 5 severe COVID-19 and *n* = 9 intermediate patients with COVID-19. (**D** and **E**) Linear regression of Horowitz index (PaO2/FiO2) or clinically measured D-dimer (μg/mL) and IL-8 score of patients with COVID-19. *P* value signifies slope significantly non-zero. 95% confidence interval shown in gray. *n* = 5 severe and 9 intermediate patients with COVID-19. (**F**) Box plot of normalized serum IL-8 plasma levels of COVID-19 and control patients. *n* = 26 control patients without COVID-19, *n* = 78 patients with mild-moderate COVID-19 (WHO Grade 1–4), *n* = 29 severe COVID-19 (WHO Grade 5–8) patients. One-way ANOVA with post hoc Tukey’s multiple comparisons test. *P < 0.05, **P < 0.01, ***P < 0.001.

Clinical and experimental data point toward a critical contribution of neutrophils to disease progression (8). To better understand how these antivirally tuned neutrophils might mediate the transition from COVID-19 pneumonia to respiratory failure we compared intermediate and severe cohorts. Biological network analyses using ClueGO yielded increased detection of proteins associated with a response to IL-8 in severe COVID-19 and a molecular skewing toward IL-8 production as the most dominant pathways in neutrophils (Figure 2A and Supplemental Figure 1F). In detail, transmembrane receptor CD74 and TLR-4, -5, and -8, which were shown to be upregulated in response to neutrophil stimulation and to trigger neutrophil IL-8 production (24), were increased in patients with severe COVID-19 (Figure 2B). We computed a global IL-8 score defined by enrichment of IL-8–associated and –induced proteins, which revealed a significant enrichment of these proteins exclusively in patients with severe COVID-19 pneumonia (Figure 2C). High IL-8 scores correlated with pulmonary disease severity of COVID-19, assessed by the Horowitz index (paO_2_/FiO_2_) (Figure 2D). In addition, we found a positive correlation between increased IL-8 scores and elevated D-dimer plasma levels, pointing toward an association of IL-8 with coagulation dysregulation (Figure 2E) (12). Hence, the neutrophil proteome signature discriminates between mild and severe courses of COVID-19. Consistent with previous reports on elevated systemic IL-8 levels, we found increased plasma IL-8 levels in patients with severe COVID-19 compared with control patients without COVID-19, in an independent cohort (*n* = 133, Figure 2F). In summary, increased ISG protein detection in neutrophils characterizes mild-to-moderate disease, whereas severe COVID-19 is associated with elevation of IL-8–induced protein pathways, corresponding with systemic increases in plasma IL-8.

### IL-8 production loops sustained by pulmonary and peripheral blood neutrophils characterize severe COVID-19.

We next hypothesized that IL-8 might be a central player involved in pulmonary recruitment of neutrophils and disease progression of COVID-19. Therefore, we analyzed publicly available scRNA-Seq data of bronchoalveolar lavage fluid from healthy controls in comparison to mild-to-moderate and patients with severe COVID-19 (25) and specifically focused on chemokine transcription in different pulmonary cell populations. Our analysis revealed the presence of IL-8 mRNA specifically in severe COVID-19 (Supplemental Figure 2, A and B), with neutrophil-like cells recruited to the alveolar space showing the strongest pulmonary IL-8 expression, followed by epithelial cells, monocytes and monocytic macrophages (Supplemental Figure 2C). Among relevant chemokines, IL-8 mRNA showed the strongest increase in neutrophils, with no elevated levels of IL-6 and IL-1alpha, and an increase in IL-1β mRNA that did not reach the same extent as IL-8 mRNA in neutrophils (Supplemental Figure 2D).

**Figure 3:**
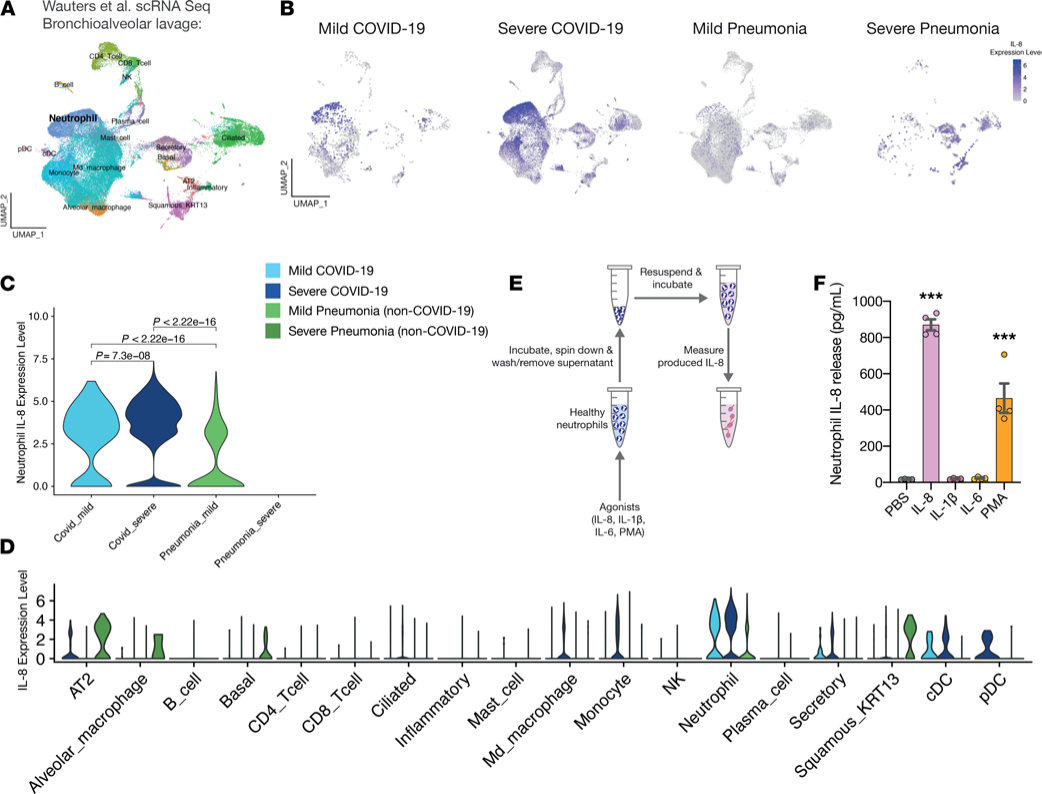
A systemic and autocrine neutrophil IL-8 loop in COVID-19. (**A** and **B**) UMAP of cells from scRNA-Seq bronchioalveolar lavage data in Wauters et al. (26). Annotated cell type in UMAP and feature plot of IL-8 expression by patient population. (**C**) Violin plot of neutrophil IL-8 expression by patient population in Wauters et al. (26) (*n* [neutrophils] = 431 for mild and *n* = 6117 for severe COVID-19, *n* = 788 for mild non–COVID-19 pneumonia and *n* = 1 for severe non–COVID-19 pneumonia). Since only 1 severe non–COVID-19 pneumonia neutrophil was detected in BALF derived from healthy patients, no violin plot depicting IL-8 production in this patient population is shown. Unpaired Student’s *t* tests. (**D**) Violin plot of IL-8 expression by cell type and patient population of Wauters et al. (26), including neutrophils. (**E**) IL-8 production assay schematic. (**F**) IL-8 production assay results. One-way ANOVA with post hoc Dunnett’s multiple comparisons test comparing IL-8 to the other conditions. *n* = 4 neutrophil donors. Mean ± SEM is shown. *P < 0.05, **P < 0.01, ***P < 0.001.

To compare COVID-19 to non–COVID-19 pneumonic patients rather than healthy controls, we additionally made use of a recently published scRNA-Seq data set by Wauters and colleagues (26), which included a high count of captured neutrophils in the dataset (Figure 3, A and B). This analysis confirmed that neutrophilic IL-8 mRNA transcription in patients with severe COVID-19 significantly surpassed expression of IL-8–encoding transcripts in both mild-to-moderate COVID-19 as well as non–COVID-19 mild pneumonia (Figure 3C). In line with our previous finding, gene expression analysis across all other detected immune and nonimmune cell types confirmed neutrophils as a main source of pulmonary IL-8 (Figure 3D).

Severe COVID-19 has been linked to cytokine shock syndromes (27–29). Recent studies revealed synergistic and self-sustaining cytokine loops as a central element of cytokine shock pathophysiology (30). Considering both our proteomic and transcriptomic analyses pointing toward IL-8 production/release by peripheral and pulmonary neutrophils, respectively, we hypothesized that a loop of neutrophil intrinsic IL-8 production with both systemic and autocrine features may drive neutrophil activation and recruitment. Therefore, we stimulated healthy neutrophils with recombinant IL-8 in vitro to mimic the systemic effects of IL-8 (Figure 3E). Interestingly, stimulation with either IL-8 or the neutrophil activating agent PMA induced IL-8 release within two hours of exposure (Figure 3F). Elevated levels of other proinflammatory cytokines like IL-1β and IL-6 in both infected lungs and plasma have been linked to COVID-19 severity (25, 31–34). However, unlike IL-8, IL-1β, and IL-6 did not lead to pronounced IL-8 release by neutrophils within the observed time frame (Figure 3F). Carryover of recombinant IL-8 was excluded in our setup using PFA-fixated neutrophils (Supplemental Figure 2E).

These data indicate a harmful cycle of neutrophil-derived pulmonary and systemic IL-8, which in turn might sustain both auto- and paracrine as well as systemic activation loops, aggravating pulmonary neutrophil influx, and local hyperstimulation.

### COVID-19 neutrophils are characterized by IL-8–induced degranulation and drive a systemic prothrombotic phenotype.

**Figure 4:**
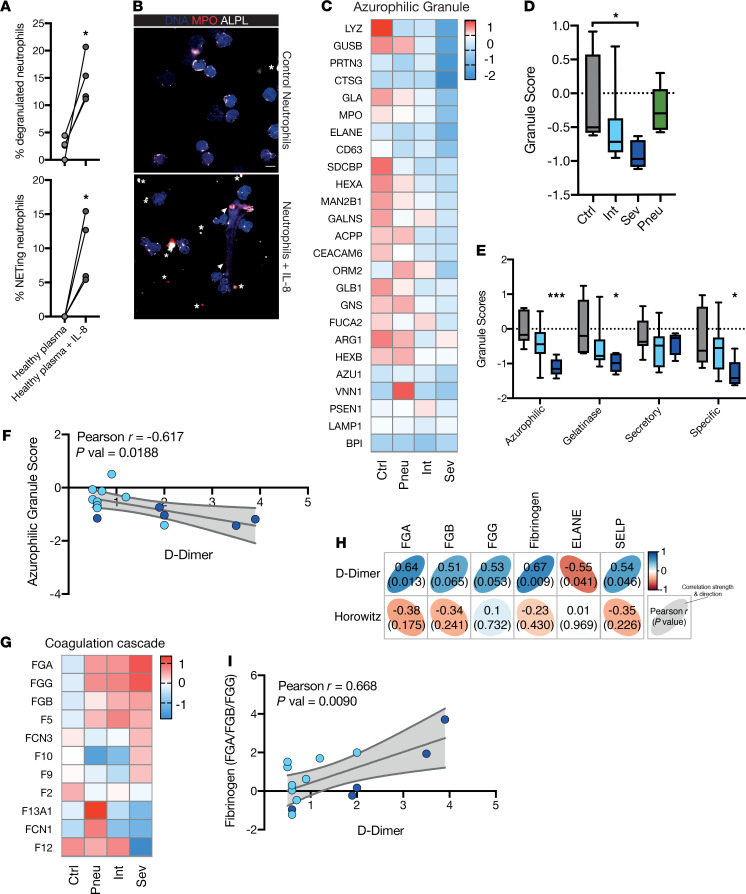
COVID-19 neutrophils are characterized by IL-8–induced degranulation and drive a systemic prothrombotic phenotype. (**A**) NETing and degranulated neutrophils as percentage of all neutrophils with control treatment of IL-8. Paired 2-sided Student’s *t* test. (**B**) Representative images of control and IL-8 treated neutrophils. Stars show degranulation, arrows NETs. Scale bar: 10 μm. ALPL, alkaline phosphatase; MPO, myeloperoxidase. (**C**) Heatmap of mean azurophilic granule protein abundance by group. (**D**) Box plot of overall granule score calculated from log-scaled abundance values (see methods) by group. One-way ANOVA with post hoc Tukey’s multiple comparisons test between all groups. (**E**) Box plots of all 4 granule scores calculated from log-scaled abundance values (see Methods) by group. Two-sided unpaired Student’s *t* test between controls and intermediate or severe COVID-19. (**F**) Linear regression of clinically measured D-dimer [μg/mL] and azurophilic granule score of neutrophil proteins of patients with COVID-19. (**G**) Heat map mean of coagulation cascade protein abundance by group. (**H**) Correlation matrix of clinically measured D-dimer and Horowitz index and neutrophil protein abundance of patients with COVID-19. Pearson r is shown in each box and as a heatmap, *P* values is in brackets. (**I**) Linear regression of D-dimer [μg/mL] and neutrophil fibrinogen protein abundance of patients with COVID-19. (**F** and **I**) *P* value signifies slope significantly non-zero. 95% confidence interval shown in gray. *n* = 5 severe and 9 intermediate patients with COVID-19. (**C–E** and **G**) *n* = 9 healthy control, *n* = 5 pneumonic controls, *n* = 5 severe COVID-19, and *n* = 9 intermediate COVID-19. *P < 0.05, **P < 0.01, ***P < 0.001.

Next, we sought to identify central effector mechanisms of IL-8–activated and –recruited neutrophils. We and others have shown a key role of NETosis in COVID-19–associated immunothrombosis (35–37). To investigate whether IL-8 might contribute to NET formation in COVID-19, we exposed neutrophils isolated from healthy donors to IL-8: IL-8 induced a marked increase in NET formation, but also led to a pronounced granule release (Figure 4, A and B). Considering the strong degranulation phenotype observed in vitro, we profiled granule contents in our COVID-19 proteome data set. Indeed, most granule proteins in primary/azurophilic (such as myeloperoxidase, MPO, and lysozyme), secondary/specific (such as CAMP), as well as tertiary/gelatinase (such as cathepsin S) and secretory vesicles (such as TREM1) were depleted from neutrophils in patients with SARS-CoV-2 pneumonia, suggesting degranulation (Figure 4C and Supplemental Figure 3, A–C) (38). In line with this result, granule scores of the major granule entities as well as a computed total granule score were decreased when comparing severe COVID-19 to healthy control patients (Figure 4, D and E). Azurophilic granules, known to contain neutrophil proteases such as Cathepsin G and Neutrophil elastase (ELANE), play a critical role in intravascular immunothrombosis (39, 40). Therefore, we examined the correlation of azurophilic granule score and D-dimer levels as surrogate parameter of systemic coagulation. Azurophilic granule score correlated negatively with D-dimer levels (Figure 4F), linking neutrophil degranulation with coagulation activation. Interestingly, RNA-sequencing data has identified increased mRNA content of granule proteins such as MPO and ELANE in immature neutrophils emerging in severe COVID-19, further supporting the notion that not a change in expression but pronounced degranulation is responsible for the observed depletion of granule proteins (21).

To identify additional neutrophil-mediated prothrombotic mechanisms, we next examined neutrophil associated coagulation and complement factors. Intermediate and severe COVID-19 neutrophils were characterized by an increased detection of multiple procoagulant proteins (Supplemental Figure 3D). Analysis of all detectable coagulation cascade proteins revealed that particularly fibrinogen (FGA, FGG, FGB), as well as coagulation factors FV, FX, and FIX were increased (Figure 4G). Both fibrinogen and the platelet marker P selectin, possibly reflecting platelet-neutrophil aggregate formation (12), correlated with the systemic prothrombotic state as assessed by D-dimer levels, whereas ELANE showed a strong inverse correlation, underlining the degranulated phenotype (Figure 4, H and I).

The complement system has been shown to contribute to both neutrophil activation, NET formation and disease progression in COVID-19, with severe cases exhibiting increases in plasma levels of both classical and alternative complement components (14, 41, 42). Neutrophils from patients with severe COVID-19 were characterized by increased detection of classical complement pathway components, such as C1R and C1S (Supplemental Figure 3, E and F). In addition, complement factors of the terminal complement complex (TCC), C5, C6, C7, C8 and C9, were significantly increased in neutrophils from intermediate and patients with severe COVID-19 (Supplemental Figure 3G). TCC formation is associated with increased platelet activation and hypercoagulability (14, 42, 43). Indeed, protein abundance of TCC and C1 on neutrophils correlated with fibrinogen on neutrophils, further indicating a role of neutrophils in driving a procoagulant phenotype in severe COVID-19 (Supplemental Figure 3, I and J). We also detected lower levels of the complement 5 receptor 1 (C5AR1/CD88) in patients with COVID-19 (Supplemental Figure 3H). Interestingly, the anaphylatoxin complement 5a (C5A) can directly bind the N-protein of SARS-CoV-2, and C5A levels have been shown to be elevated in critical COVID-19 (41). Given that C5A binding to neutrophils can trigger hyperactivation (44), the reduction of C5AR1 observed in our dataset may be due to C5A-induced degranulation and subsequent cleavage of C5AR1 from the neutrophil surface (45).

In summary, our data highlight multiple prothrombotic pathways in severe COVID-19 neutrophils — namely (a) NETosis, (b) direct engagement of the coagulation and (c) complement cascade, and (d) enhanced granule release. In addition, our in vitro findings link both neutrophil degranulation and NET formation to IL-8 exposure. This suggests that the high levels of IL-8 detected in plasma of patients with severe COVID-19 (7, 8, 46) may play a pivotal role in driving a prothrombotic neutrophil phenotype.

### Therapeutic blockade of IL-8 reduces COVID-19–associated neutrophil activation in vitro and attenuates ARDS-related microthrombosis in vivo.

**Figure 5:**
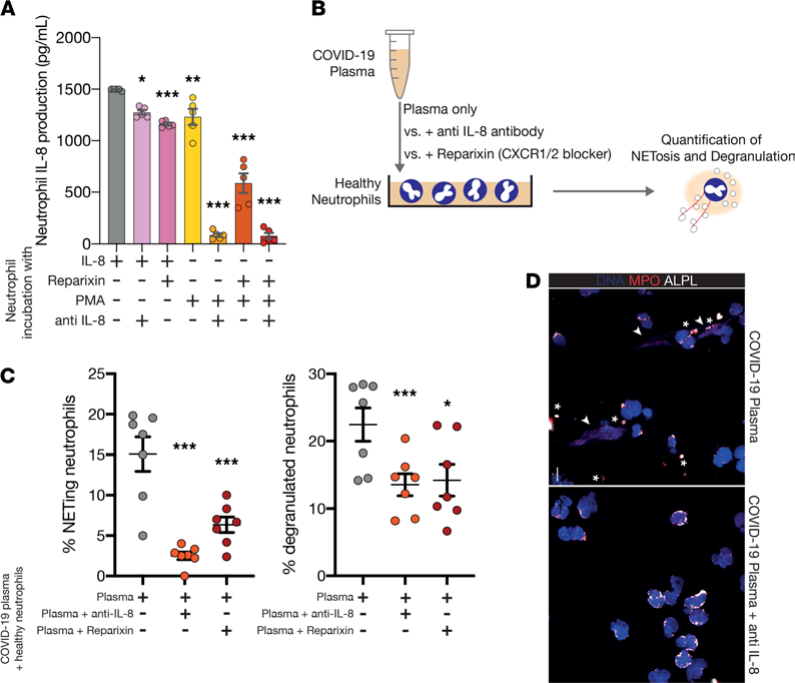
Therapeutic blockade of IL-8 reduces COVID-19–associated neutrophil activation in vitro. (**A**) IL-8 production and blocking assay similar to Figure 2E. One-way ANOVA with post hoc Dunnett’s multiple comparisons test comparing IL-8 to the other conditions. *n* = 5 healthy neutrophil donors. (**B**) Illustration of in vitro experiment: Neutrophils from healthy donors were exposed to plasma from patients with severe COVID-19 and pretreated with vehicle, anti–IL-8 antibody or the CXCR-1/-2 antagonist reparixin. Degranulation, NETosis and surface expression of activation markers were assessed by microscopy and flow cytometry, respectively. (**C**) NETing and degranulated neutrophils as percent of healthy neutrophils stimulated with COVID-19 plasma and nothing, anti–IL-8 antibodies or reparixin. Paired 2-sided Student’s *t* test, *n* = 7 plasma samples from patients with COVID-19. (**D**) Representative images of COVID-19 plasma and COVID-19 plasma and anti–IL-8 incubated neutrophils. Stars show degranulation, arrows NETing. Scale bar: 10 μm. *P < 0.05, **P < 0.01, ***P < 0.001.

Based on the above findings, we hypothesized that IL-8*–*induced signaling may be one of the culprit pathways for SARS-CoV-2–induced neutrophil activation and recruitment loops that drive neutrophil-induced lung injury. Therefore, we assessed the possibility to therapeutically interfere with autocrine IL-8 production in neutrophils. To this end, we isolated neutrophils from healthy donors, stimulated them and assessed IL-8 production in response to either IL-8 or the NET-inducing agent PMA (methods see Figure 3E). The effect of both IL-8 and PMA on autocrine IL-8 secretion was reduced when incubating neutrophils with either an anti–IL-8 antibody or reparixin, a clinically available, oral IL-8 receptor (CXCR-1/-2) blocker (47, 48), suggestive of autocrine, self-sustaining IL-8 production loops in neutrophils (Figure 5A).

Next, we exposed neutrophils derived from healthy donors to plasma isolated from either healthy donors or patients with COVID-19 pneumonia (Figure 5B). COVID-19 plasma induced neutrophil activation even after a short incubation period, that could be partially attenuated by IL-8 blockade (Supplemental Figure 4, A and B). Correspondingly, neutrophil degranulation and NET formation induced by COVID-19 plasma were reduced upon blockade of IL-8 signaling using either an anti–IL-8 antibody or reparixin (Figure 5, C and D). In fact, blocking IL-8 in COVID-19 plasma with a neutralizing, monoclonal anti–IL-8 antibody reduced COVID-19 induced NETosis in neutrophils by over 80% (Figure 5C).

**Figure 6:**
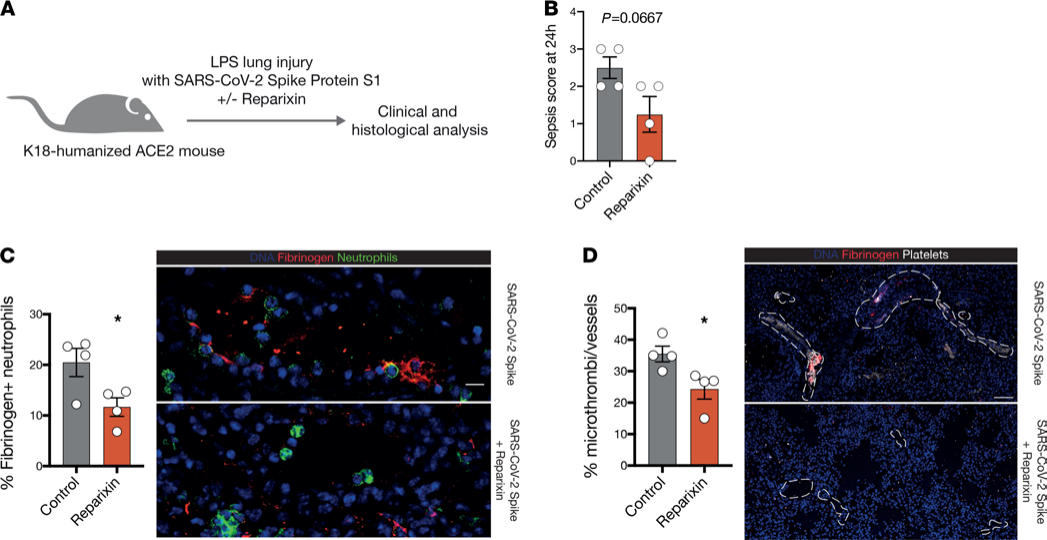
Therapeutic blockade of IL-8 signaling attenuates ARDS-related microthrombosis in vivo. (**A**) Illustration of in vivo SARS-CoV-2 S1 spike protein–induced lung injury mouse model. (**B**) Clinical sepsis score at 24 hours after S1 spike protein–induced lung injury for control and reparixin-treated hACE2 mice. (**C**) Quantification and representative micrographs of fibrinogen-positive/fibrinogen-binding neutrophils in one vessel in the lungs of control or reparixin-treated mice. Scale bar: 10 μm. (**D**) Quantification and representative micrographs of microthrombi in lungs of vehicle or reparixin-treated mice. Vessel borders are shown with dashed white lines. Scale bar: 50 μm. (**B–D**) Two-tailed unpaired Student’s *t* test, *n* = 4 per group. Data are shown as the mean ± SEM. *P < 0.05, **P < 0.01, ***P < 0.001.

Finally, to confirm our results in vivo, we employed a mouse model of COVID-19-associated respiratory failure (49). In transgenic mice expressing the human ACE2 (hACE2) receptor, intranasal exposure to LPS and recombinant SARS-CoV-2 spike protein led to pulmonary microthrombosis, mimicking immunothrombosis observed in human lungs (Figure 6A and Supplemental Figure 4D) (12, 50). When comparing hACE2 negative mice with hACE2 expressing littermates, clinical severity and pulmonary microthrombosis were enhanced in hACE2-expressing mice treated with both LPS and SARS-CoV-2 spike protein (Supplemental Figure 4, E and F) (50). This effect is dependent on hACE2 expression and spike protein binding, as hACE2 expressing littermates challenged with LPS, without spike protein, did not show enhanced microthrombosis or increased clinical severity (Supplemental Figure 4, E and F), confirming spike protein–mediated pulmonary endotheliopathy (51). In patients with COVID-19, neutrophils binding of fibrinogen correlated positively with parameters of coagulation activation assessed by D-dimer (Figure 4I). Indeed, the number of fibrinogen-binding intravascular neutrophils was also increased in this mouse model, specifically for spike protein–treated hACE2-positive animals (Supplemental Figure 4G). Although IL-8 itself is not expressed in mice, its homologues CXCL-1, CXCL-2, and GCP-2/CXCL-6 signal via the same receptor pair (CXCR-1/-2) and exert similar functions (52, 53). Blocking IL-8–like signaling with reparixin resulted in a trend toward clinical improvement of hACE2 mice at 24 h, as compared with control animals (Figure 6B and Supplemental Figure 4I). Reparixin also reduced fibrinogen binding by intravascular neutrophils (Figure 6C). Finally, we observed that spike protein–induced pulmonary microthrombosis was significantly attenuated by reparixin treatment in this mouse model of COVID-19 immunopathology (Figure 6D and Supplemental Figure 4H).

## Discussion

Immunopathology caused by dysregulated cellular responses in life-threatening COVID-19 has been shown to contribute to local and remote organ damage (9). Moreover, dysregulation of the myeloid compartment, characterized by the emergence of immature neutrophil populations in peripheral blood, along with a global skewing toward a procoagulant neutrophil state lead to thrombo-occlusive events (7–9, 21, 32, 33, 54, 55). Uncontrolled release of cytokines by innate immune cells might drive these self-destructive, immunopathological loops, but mechanistic studies to highlight possible therapeutic targets remain scarce (7, 32, 33, 56).

Using mass-spectrometric analyses, we provide a deep proteome of COVID-19 neutrophils. Detecting more than 5000 proteins, our data allow for a broad understanding of neutrophil biology in this disease; in contrast to RNA sequencing, this neutrophil proteome also enables analysis of protein binding and release and reflects the actual functional state of the cells in vivo. This is specifically of interest considering previous reports showing that in neutrophils, transcript abundance may not necessarily correlate with respective protein levels (57). This observation is also highlighted in our data set, in which we observe depletion of granule proteins in severe COVID-19, whereas prior work using RNA sequencing has described increased granule protein-encoding transcripts (21). These diverging results can be reconciled by granule release, which cannot be captured by sequencing technology. We identify additional severe proteomic perturbations in neutrophils from patients with COVID-19: Increased ISG products in neutrophils from patients with COVID-19 with mild-to-moderate severity indicate an antiviral neutrophil response to SARS-CoV-2. In severe COVID-19, this initially protective response may deteriorate into a hyperactivated and procoagulant phenotype distinct from other viral pneumonias: When comparing moderate disease and respiratory failure, we uncovered a dominant proteomic signature indicative of IL-8 production and signaling. Through analysis of bronchoalveolar lavage scRNA-Seq data and in vitro experiments, we confirmed IL-8 production by both pulmonary and circulating neutrophils, which might explain their mobilization and influx into the failing lung. Of note, IL-8 mRNA transcription was accentuated in alveolar neutrophils from severely ill patients, and in vitro incubation of neutrophils with IL-8 in turn triggered prominent IL-8 release. Other cytokines like IL-1β and IL-6, which have been linked to COVID-19 severity and the ensuing cytokine storm (27, 30–32), did not exhibit comparable increases in pulmonary neutrophils and did not elicit IL-8 release from blood neutrophils in vitro. These data indicate systemic and autocrine IL-8 loops to be central to neutrophil-driven immunopathology. Of note, this pathway does not explain the phenotype of intermediate severity neutrophils, suggesting the involvement of additional signaling cascades. Focusing on critical disease, recent studies have identified a strong link between circulating IL-8 plasma levels and respiratory failure as well as SARS-CoV-2–associated mortality (8, 32). We confirm these findings by showing that plasma IL-8 levels are selectively elevated in severe, but not in mild-to-moderate COVID-19 or non–COVID-19 patients.

We further investigated how neutrophils might mediate their prothrombotic and immunopathological potential in COVID-19. We identified multiple so far unrecognized prothrombotic pathways linking neutrophil activation to hypercoagulability: In addition to NET formation and platelet-neutrophil interplay, we highlight increased detection of coagulation and complement factors, as well as neutrophil granule release as key aspects of severe COVID-19. These data are in line with previous observations of both increasing formation of aggregates consisting of neutrophils and activated platelets as well as binding/production of complement factors by neutrophils and complement-driven immunothrombosis in COVID-19 (12, 14, 58).

We therefore hypothesized that targeting IL-8 may interfere with this vicious cycle of neutrophil activation and prothrombotic phenotype (37). Here, we show that anti–IL-8 antibodies and targeted inhibition of IL-8 signaling by the CXCR-1/-2 blocker reparixin reduced neutrophil activation, degranulation and NET formation caused by COVID-19 plasma. To validate our findings in vivo, we established a novel model of SARS-CoV-2 spike protein and LPS-induced acute lung injury that mimics previous findings from human disease, in particular an increase in pulmonary microthrombi and procoagulant neutrophils. Given that this model does not require extensive safety requirements compared with live SARS-CoV-2 or other proxy viruses, our spike protein–dependent model may serve as versatile experimental basis for further COVID-19-related research. Notably, blocking IL-8–like signaling through CXCR-1/-2 decreased disease severity in our murine model of SARS-CoV-2–associated acute respiratory distress syndrome (ARDS). Confirming our insights from the human neutrophil proteome, we observed decreased fibrinogen binding by neutrophils in reparixin-treated animals and an attenuation of pulmonary microthrombosis. These findings are particularly noteworthy in light of recent phase 2/3 clinical trials that investigate the use of reparixin in moderate and severe COVID-19 pneumonia (e.g., NCT04794803, now NCT04878055). In addition, we provide evidence for spike protein–induced increases in both clinical severity, neutrophil activation and microthrombosis. The mechanisms by which addition of spike protein alone exacerbates acute lung injury in absence of live virus and replication-associated cytolysis remain to be investigated. Notably, binding of coronavirus spike protein to its receptor hACE2 (59) has been shown to result in downregulation of hACE2 surface expression and hACE2 shedding for both SARS-CoV-1 (60) and SARS-CoV-2 (51). Given that hACE2 is known to attenuate acute lung injury in mouse models of SARS-CoV-1 mediated ARDS (49, 61), reduced local hACE2 availability and subsequent hyperactivation of angiotensin II–mediated (ATII-mediated) signaling may represent a possible mechanism of spike protein–induced endothelial dysfunction and aggravation of pulmonary damage (51).

In summary, we provide insights into the molecular mechanisms of IL-8–mediated immunopathology in COVID-19 that lay out a promising basis for therapeutic targeting, specifically in patients progressing to severe COVID-19.

## Methods

### Cohort.

A total of 40 patients were included in our study (*n* = 22 patients with RT-PCR–confirmed COVID-19 and *n* = 18 control patients without COVID-19). For proteomics analysis we recruited a total of 28 patients. Of these, 14 were hospitalized, 5 of whom required invasive ventilation (CoV_sev); all CoV_sev patients met the Berlin Criteria for ARDS (62). In addition, 5 patients with viral pneumonias and 9 healthy controls were included. Details of our cohort are described in Nicolai et al. (12). For in vitro validation experiments, 7 patients with COVID-19 (4 male patients, mean age 65) and 5 healthy volunteers were recruited. Patients with COVID-19 were divided into a group of severe cases requiring intubation and intensive care treatment (CoV_sev, *n* = 11), and a group of intermediate severity (CoV_int, *n* = 11), which were hospitalized. Control patients were divided into non-pulmonary controls (Ctrl, *n* = 13), and patients with other viral pneumonias (Ctrl_pneu, *n* = 5). Horowitz indices (paO_2_/FiO_2_) of CoV_sev patients were derived from ventilation parameters or approximated (12).

For assessment of plasma IL-8 levels, samples from a total of 133 patients, who were admitted to LMU University Hospital in Munich for suspected or confirmed COVID-19 were included (Patient characteristics in Supplemental Table 4). Plasma samples were measured using a Proximity Extension Assay (PEA, Olink Proteomics) as described previously (63).

### Neutrophil preparation from whole blood.

Patient blood was mixed with a Lysing-PFA-Fixation solution containing Heparin, distilled water, and FACS Lysing Solution (BD). Lysates were centrifuged and frozen. Samples were thawed, spun down, and stained. After adding staining, neutrophils were sorted (Gating: Singlets>Size>CD45+>SSC vs CD15>Neutrophils). More than 80,000 neutrophils were sorted per sample. After sorting, neutrophils were resuspended in 2% SDS in 50 mM TRIS Buffer (pH 8) and boiled at 95°C and 100 rpm for 30 minutes.

### Sample preparation for mass spectrometry and analysis.

For proteomics analysis, samples were boiled in SDS buffer and subjected to SP3-based cleanup and tryptic digest (64). To enable multiplexing, samples were labeled with TMTpro 16-plex reagents (Thermo Fisher Scientific) (65). A total of 5 μg peptide/sample was assigned to channels 1–12. Pooled plexes were subjected to high-pH HPLC separation into 24 fractions. An estimate of 1 μg each fraction was measured on a Q Exactive HF-X mass spectrometer (Thermo Fisher Scientific). For database search, MaxQuant version 1.6.10.43 (66) was used while enabling TMTpro 16-plex reporter ion quantitation with a PIF setting of 0.5. Downstream analysis was done with R. For quantitation, a minimum of 75% valid TMT reporter ion intensities was required. The remaining missing values were imputed by employing k-nearest neighbor algorithm. Corrected reporter ion intensities were normalized against the internal reference sample and scaled using median-median absolute deviation (median-MAD) normalization. For significance calling 2-sample moderated, 2-tailed Student’s *t* testing as well as moderated F testing was applied (limma R package) (67). *P* values were adjusted using the Benjamini-Hochberg method.

### Neutrophil activation with patient plasma.

Neutrophils were isolated using the EasySep Neutrophil Isolation Kit (Stemcell Technologies), counted, and resuspended in RPMI containing L-glutamine and 5% FCS. Neutrophils were exposed to plasma (10% final concentration) derived from healthy donors (*n* = 4) or patients with COVID-19 (*n* = 7) and subsequently stained for indicated activation markers. For selected subgroups, human IL-8 (MilliporeSigma, I1654), anti-human IL-8 antibody (MilliporeSigma, I2519), or reparixin (20 μM, SelleckChem, S8640) were added to either plasma or neutrophils 20 minutes before neutrophil exposure to plasma. Cells were incubated at 37°C and 5% CO_2_ for 1 hour, fixated using 1% PFA. Mean fluorescent intensities (MFIs) of neutrophils (singlets>size>CD15^+^+CD16^+^) were assessed.

In a separate series of experiments, isolated healthy neutrophils were added to poly-L lysine coated Ibidi μ-slides and treated with plasma (20% final concentration) and/or inhibitors as described above. Neutrophil granules, released vesicles and NETS were stained using antibodies against myeloperoxidase (MPO, R&D Systems, AF3667) and neutrophil alkaline phosphatase (ALPL, MilliporeSigma, HPA008765) and secondary antibodies (anti-goat AF594, anti-rabbit AF647, 1:200, Invitrogen) along with 4′,6-diamidin-2-phenylindole (1:1000) and SytoxGreen (Thermo Fisher Scientific, S7020, 500 nM final concentration, see Supplemental Figure 4C).

### Assessment of neutrophil IL-8 secretion in vitro.

Isolated neutrophils (*n* = 5) were exposed to human IL-8 (20 ng/mL, MilliporeSigma I1654), human IL-1β (20 ng/mL), human IL-6 (20 ng/mL, BioLegend 570804) or phorbol myristate acetate (PMA, 100 nM, EnzoLifeSciences, BML-PE160-0001) for 1 hour at 37°C. Subsequently, neutrophils were washed twice, spun down, resuspended in RPMI, and incubated for 2 hours. RPMI contained either an IL-8 blocking antibody (10 μg/mL), reparixin (20 μM), or both. In some experiments, neutrophils were fixated with 4% PFA to ensure that measured IL-8 levels in nonfixated samples reflected secretion and were not simply a carryover of recombinant IL-8. Stimulated neutrophils were spun down, and supernatant was collected and snap-frozen on dry ice. IL-8 content of neutrophil supernatants was assessed using an IL-8 ELISA kit (Abcam, ab46032) and a plate reader (450 nm, Tecan Infinite F200).

### In vivo model of SARS-CoV-2 S-protein and LPS-induced ARDS.

Mice transgenic for the human ACE2 receptor (K18-hACE2, B6.Cg-Tg(K18-ACE2)2Prlmn/J, 034860, The Jackson Laboratory) were exposed to 20 μg LPS (MilliporeSigma) or LPS 20 μg and 10 μg SARS-CoV-2 S1 spike protein (Abcam) i.n. to establish the model of spike protein–induced pulmonary injury. hACE2-negative litter mates served as controls. In a separate series of experiments, hACE2-positive mice were treated with both LPS and spike protein applied i.n., with one group of animals receiving reparixin (15 μg/g BW i.p.) 1 hour before and 2 hours after LPS/spike protein challenge (50, 68). Clinical scoring was performed by a scientist blinded to treatment. Mice were sacrificed after 24 hours, and lungs were removed and either processed for histopathological analysis (see below).

### Histopathological analysis of murine lungs.

Lung slices were fixated and stained using primary antibodies targeting Ly6G (BioLegend, 127632), MPO (R&D Systems, AF3667), CD42b (Abcam, 183345), and Fibrinogen (R&D Systems, AF4786) (12). Stained lung slices were imaged on an inverted Zeiss LSM 880 confocal microscope in AiryScan Super Resolution (SR) Mode (magnification, ×20/0.8 obj.), with 5–6 random images acquired per lung.

### Analysis of scRNA-Seq data from bronchoalveolar lavage fluid.

Count matrices for the scRNA-Seq dataset published by Liao and colleagues (25) as well as Wauters et al. (26) were downloaded from GEO (accession GSE145926) and the EGA European Genome-Phenome Archive database (EGAS00001004717), respectively. scRNA-Seq data were analyzed using Seurat 3.1.1 (https://github.com/mjoppich/CovidImmune/commit/977e60da8a6b4c03f628193f6348a27a65ba4578; branch, https://github.com/mjoppich/CovidImmune/tree/jci). A table with annotated cell types and their absolute prevalence is shown in Supplemental Tables 2 and 3. For cell type–specific analysis, neutrophils were defined according to expression of population-defining gene sets in the Liao et al. data set (e.g., CD16/FCGRIII, S100A9), whereas neutrophils in the scRNA-Seq data from Wauters et al. were identified as described by the authors (26).

### Data sharing statement.

Processed proteomic data are available for quick visual insights online on shiny MDC (https://neuprocov.mdc-berlin.de/). This includes graphical interfaces and a protein expression table download to compare neutrophil protein dynamics in healthy patients, viral pneumonia, and SARS-CoV-2 pneumonias. For all other original data, please contact the corresponding authors.

### Statistics.

Data were analyzed using Excel v16 (Microsoft), Prism v8.3.0. (GraphPad) and R v4.0.3. For direct comparisons between 2 groups, unpaired or paired 2-tailed Student’s *t* tests were used, as indicated. For comparisons between groups 1-way ANOVAs with Dunnett’s post hoc tests were used. Only post hoc test results are displayed in the graphs. *P* values of less than or equal to 0.05 are considered significant. For proteomic data, the adjusted *P* value was used to determine significance in case of singular protein abundance comparisons. For pathway analysis Cytoscape 3.8.1 with ClueGo 2.5.7 were used (69, 70). The top 100 differentially expressed proteins were used, with GO-BP pathways with a *P* value of less than 0.05 and medium network specificity shown. The network is displayed by significance of the groups’ individual pathways, with arrows signifying direction of association between pathways. The network term shown is the one that is the most significant of its group. Individual patient data are represented as dots, unless otherwise indicated. Protein scores were computed as the mean of the median-MAD column based on normalized protein abundance (compare Supplemental Table 1). Scores were determined after an extensive literature search: For granule scores, Adrover et al. (38) was used as a reference, for procoagulant and complement groups, all detectable coagulation cascade factors and complement factors were included (71–75). For IL-8–related proteins, a literature search for detectable relevant pathway proteins was used for the score (76–88). For regression analyses Prism or R with corrplot (89) were used. Gray lines represent best-fit line, and the gray area the 95% confidence interval. Pearson r and *P* values (test for slope non-zero) are shown in plots.

### Study approval.

In accordance with the Declaration of Helsinki, informed consent of the patients or guardians was obtained. Patients with COVID-19 and included control patients are part of the COVID-19 Registry of the LMU University Hospital Munich (CORKUM, WHO trial ID DRKS00021225). Pseudonymized data were used for analysis, and the study was approved by the ethics committee of LMU Munich (no. 20-245 and 19-274).

## Author contributions

AL, LN, and KP initiated the project. AL, LN, KP, and KS conceptualized the project. RK, AL, KP, OP, MJ, PM, and LN contributed to the methodology. RK, AL, KP, OP, MJ, VP, MLH, CG, SB, AE, RE, AA, AT, SS, VK, AT, AH, LS, and LN contributed to all investigations. MM, JCH, CS, KS, RZ, AH, CS, SK, NH, SM, PM, and KS provided all resources. AL, RK, KP, RE, AA, OP, PM, and LN conducted formal analysis. RK, AL, KP, LN, and KS wrote the original draft. All authors edited the revision. AL, OP, and MJ provided data curation and software. AL, OP, MJ, and RK performed visualization. LN, AL, RK, KP, RZ, PM, and KS supervised and performed project administration. LN, KP, SM, PM, and KS managed funding acquisition. Equally contributing authors are listed alphabetically.

## Supplementary Material

Supplemental data

## Version 1 08/17/2021

In-Press Preview

## Version 2 09/22/2021

Electronic publication
