# Supplementary material for: Self-sustaining IL-8 loops drive a prothrombotic neutrophil phenotype in severe COVID-19
Source: JCI Insight. 2021 Sep 22;6(18):e150862. doi: 10.1172/jci.insight.150862 (PMC8492337; doi:10.1172/jci.insight.150862)
Supplement: Supplemental data [file jciinsight-6-150862-s268.pdf]

Supplemental Information

Self-sustaining interleukin-8 loops drive a prothrombotic neutrophil phenotype in severe COVID-19

Rainer Kaiser[1,2,3*], Alexander Leunig[1,2,*,§], Kami Pekayvaz[1,2,3*], Oliver Popp[4,5], Markus Joppich[6], Vivien Polewka[1], Raphael Escaig[1], Afra Anjum[1], Marie-Louise Hoffknecht[1], Christoph Gold[1], Sophia Brambs[1], Anouk Engel[1], Sven Stockhausen[1,2], Viktoria Knottenberg[1], Anna Titova[1], Mohamed Haji[4,5], Clemens Scherer[1,2,3], Maximilian Muenchhoff[3,8,9], Johannes C. Hellmuth[3,10], Kathrin Saar[4,5], Benjamin Schubert[11,12,13], Anne Hilgendorff[13,14,15], Christian Schulz[1,2], Stefan Kääb[1,2,3], Ralf Zimmer[6], Norbert Hübner[4,5,7], Steffen Massberg[1,2,3], Philipp Mertins[4,5], Leo Nicolai[1,2,3,#,§], Konstantin Stark[1,2,3,#]

Affiliations:

1 Department of Medicine I, University Hospital, Ludwig-Maximilians University Munich, Germany
2 DZHK (German Centre for Cardiovascular Research), partner site Munich Heart Alliance, Germany
3 COVID-19 Registry of the LMU Munich (CORKUM), University Hospital, Ludwig-Maximilians University Munich
4 Max Delbrück Center for Molecular Medicine (MDC) in the Helmholtz Association, Berlin, Germany
5 DZHK (German Centre for Cardiovascular Research), partner site Berlin, Germany
6 Department of Informatics, Ludwig-Maximilians-Universität München, Munich, Germany
7 Charité-Universitätsmedizin Berlin, Germany
8 Max von Pettenkofer Institute & GeneCenter, Virology, Faculty of Medicine, Ludwig-Maximilians University, Munich, Germany
9 German Center for Infection Research (DZIF), Partner Site Munich
10 Medizinische Klinik und Poliklinik III, University Hospital, Ludwig-Maximilians University Munich, Germany
11 Institute of Computational Biology, Helmholtz Zentrum München—German Research Center for Environmental Health, Neuherberg 85764, Germany
12 Department of Mathematics, Technical University of Munich, Garching bei München 85748, Germany
13 On behalf of the COMBAT C19IR study group
14 Institute for Lung Biology and Disease and Comprehensive Pneumology Center with the CPC-M bioArchive, Helmholtz Center Munich, Member of the German Center for Lung Research (DZL), Munich, Germany
15 Center for Comprehensive Developmental Care (CDeC[LMU]) at the interdisciplinary Social Pediatric Center, Haunersches Children`s Hospital, University Hospital Ludwig-Maximilian University, Munich, Germany

* Contributed equally, ordered alphabetically
# Contributed equally, ordered alphabetically
§ Correspondence to: Alexander.Leunig@med.uni-muenchen.de, Leo.Nicolai@med.uni-muenchen.de, Medizinische Klinik und Poliklinik I, University Hospital Ludwig-Maximilians University Munich, Marchioninistr. 15, 81377, Munich, Germany. Tel.: +49 89 2180 76582 Fax: +49 89 2180 76583.

Supplemental Figures

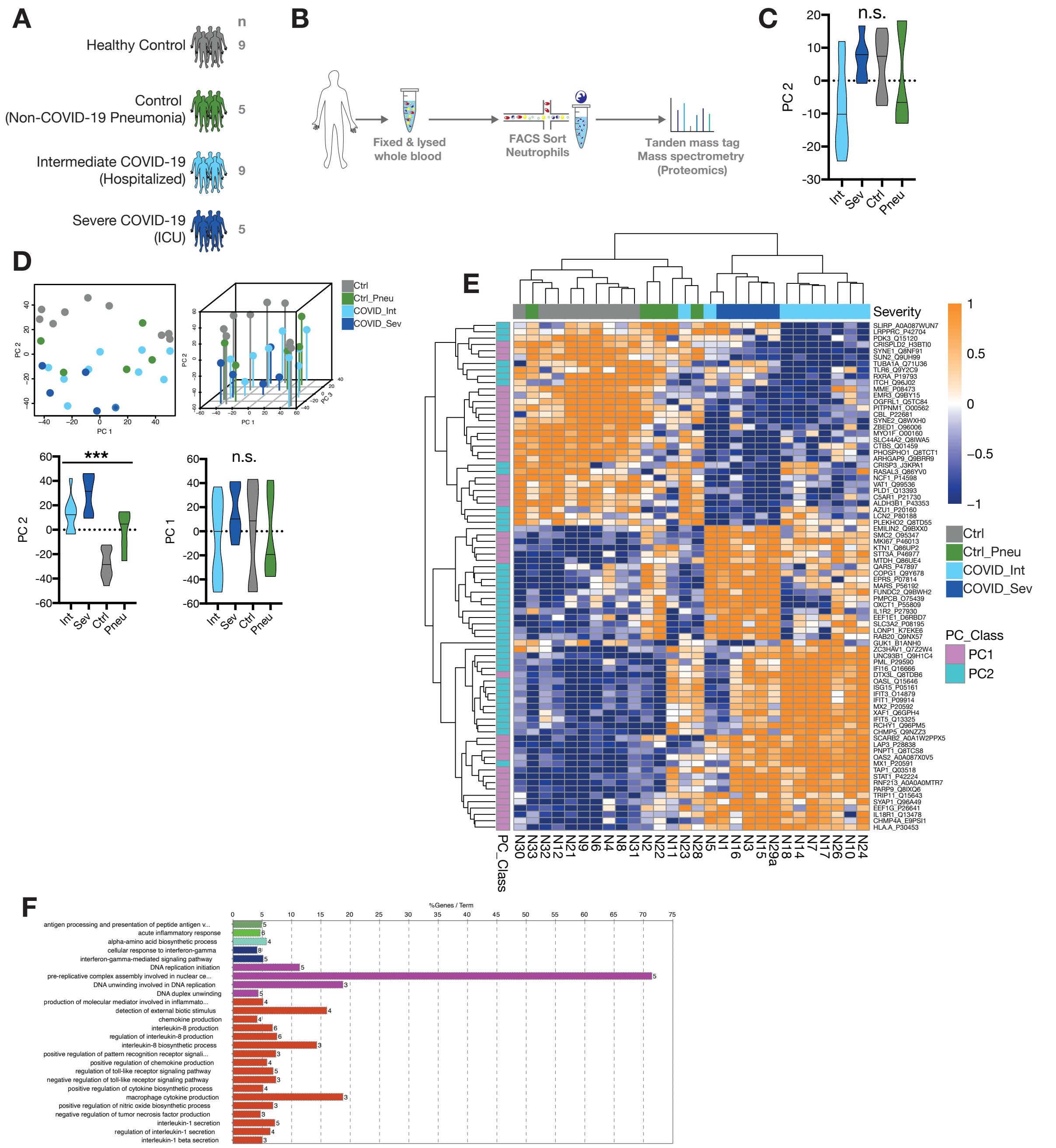

Suppl Figure 1 | a-b, Patient population and workflow for neutrophil proteomic **c,** Positions of patient neutrophil samples on PC 2 of **Fig. 1a**. One-way ANOVA. **d,** Principal component analysis of neutrophils of patients, by group. All proteins included. Violin plots of positions of patient neutrophil samples on PC 1&2. One-way ANOVA. **e,** Top 20 PCA loadings for PC1 and PC2 of a moderated F-test over all four patient groups. **f,** ClueGo upregulated neutrophil proteome functional network and chart of severe COVID-19 compared to intermediate COVID-19 (see methods). Full ClueGo term names are shown in Suppl. Table 5. **a-f:** n=9 healthy control, n=5 pneumonic control, n=5 severe COVID-19 and n=9 intermediate COVID-19 patients. *p<0.05, **p<0.01, ***p<0.001.

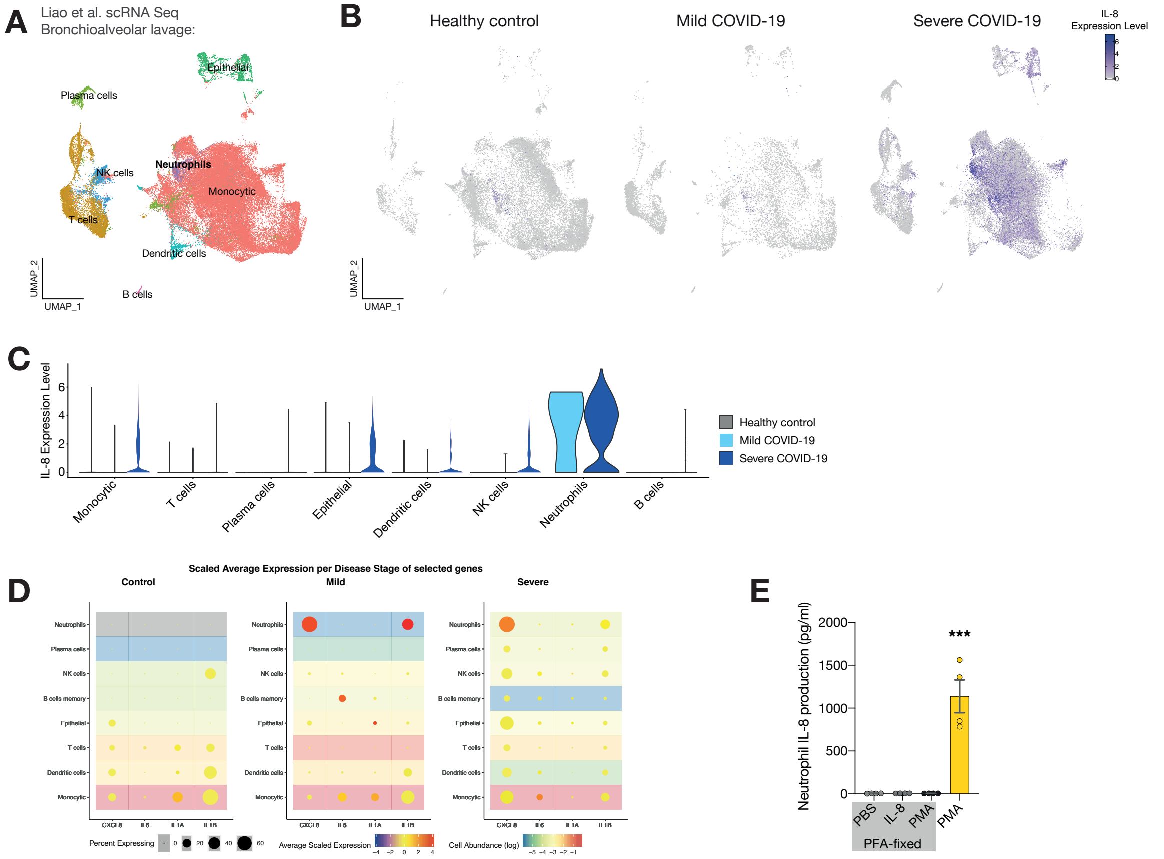

Suppl. Figure 2 | a-b, UMAP of cells from scRNA-seq bronchioalveolar lavage data of Liao et al. (see ref. 25). Annotated cell type in UMAP and feature plot of IL-8 expression by patient population. **c,** Violin plot of IL-8 expression by cell type and patient population. n [neutrophils] = 3 for intermediate and 807 for severe COVID-19. Since no neutrophils were detected in BALF derived from healthy patients, no violin plot depicting IL-8 production in these cells is shown. **d,** Dot-plot of the scaled average expression of CXCL (IL-8), IL-6, IL-1alpha and IL-1beta from scRNA-seq bronchioalveolar lavage data of Liao et al. **e,** IL-8 production assay results with either 4% PFA-fixed or unfixed neutrophils and the addition of PBS, IL-8 or PMA. One-way ANOVA with post-hoc Dunnett's multiple comparisons test comparing IL-8 to the other conditions. n=4 neutrophil donors. Mean ±sem is shown. *p<0.05, **p<0.01, ***p<0.001.

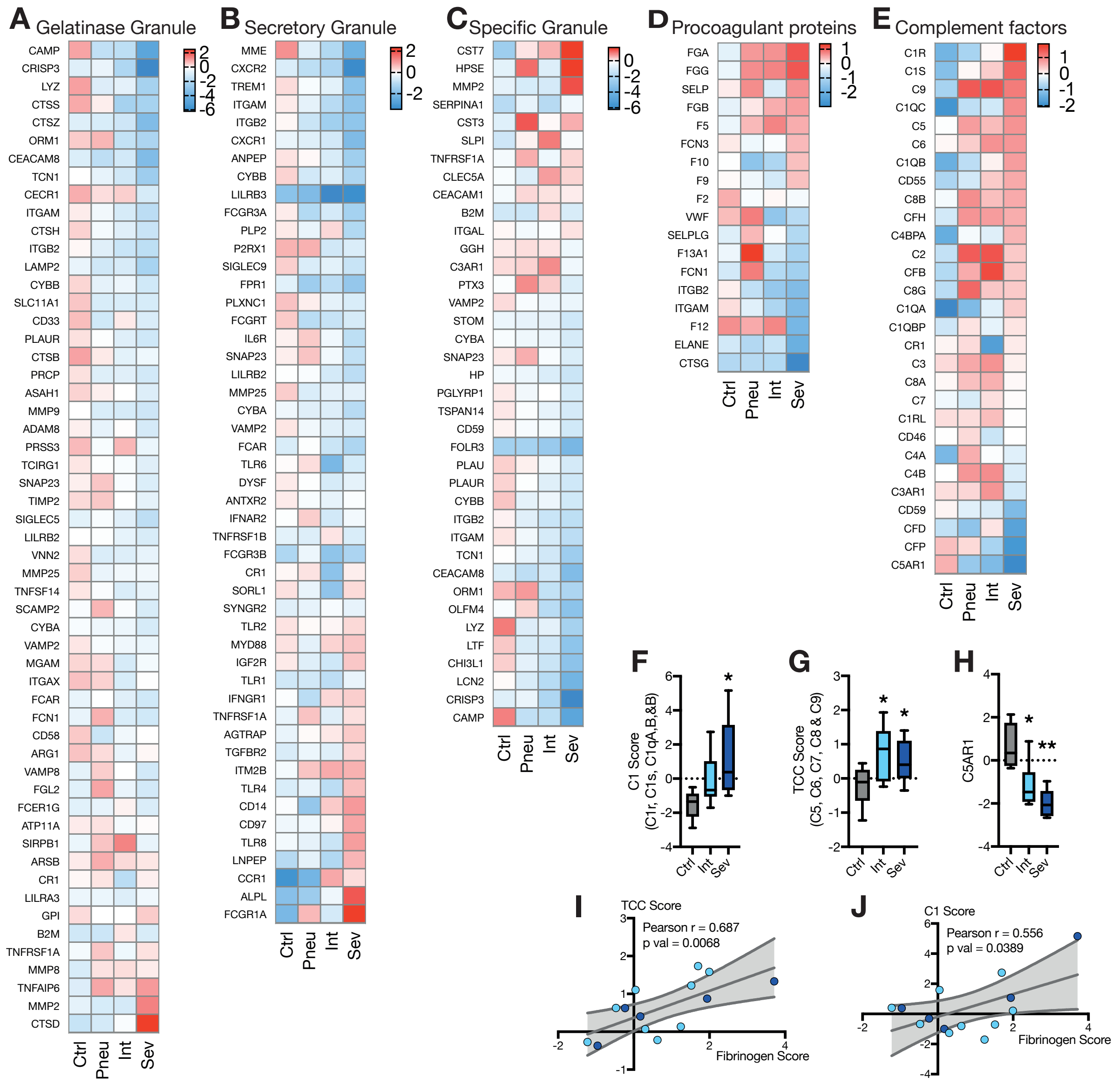

Suppl. Figure 3 | a-c, Heat maps of mean gelatinase/primary, secretory/secondary and specific/tertiary granule protein abundance by group. **d,** Heat map of mean procoagulant proteins abundance by group. **e,** Heat map of mean complement factor protein abundance by group. **f-g,** Box plot of Terminal complement complex (TCC) and C1 score by group. Unpaired, two-sided t-tests comparing to control. **h,** Box plot of C5AR1 abundance by group. Adjusted p-values. **i-j,** Linear regression of fibrinogen protein abundance on neutrophils and terminal complement complex or C1 protein abundance on neutrophils. Pearson r is shown, p-value signifies slope significantly non-zero. 95% confidence interval shown in gray. n=5 severe and 9 intermediate COVID-19 patients. *p<0.05, **p<0.01, ***p<0.001.

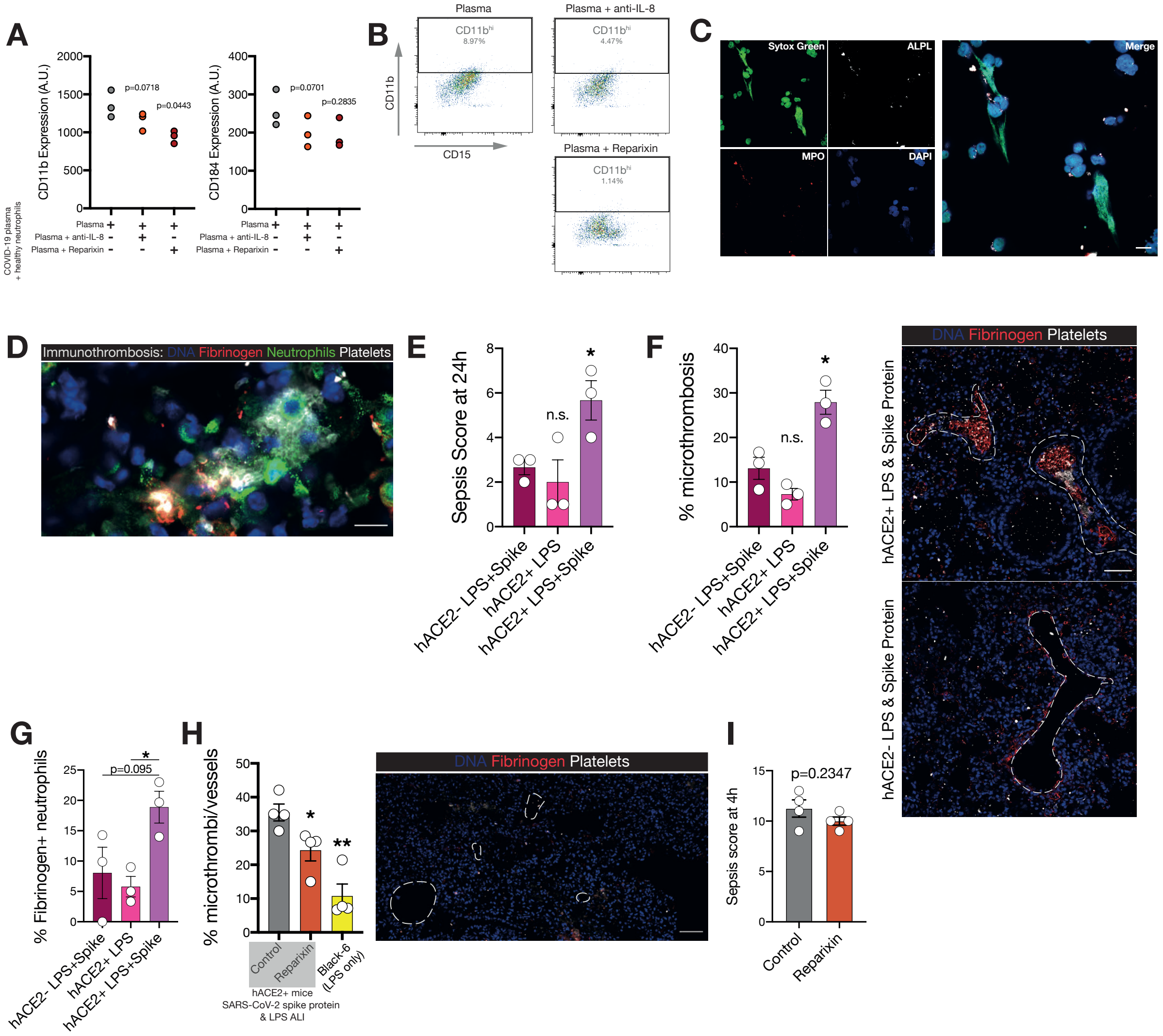

Suppl. Figure 4 | a, Surface marker expression (CD11b & CD184) of healthy neutrophils stimulated with COVID-19 plasma and PBS, anti-IL-8 antibodies or reparixin. Paired two-sided t-test, n=3 COVID-19 patient plasma samples. **b,** Illustrative flow-cytometry plots of healthy neutrophils stimulated with COVID-19 plasma and PBS, anti-IL-8 antibodies or reparixin. Percentage of positive cells shown in gate. **c,** Separate channels and merge of image from **4d**. Sytox green channel for NET identification is also shown. Scale bar 10 µm. **d,** Micrograph of immunothrombotic occlusion in one vessel observed in the lung of hACE2 SARS-CoV-2 Spike protein lung injury mice. Scale bar 10µm. **e-f,** Clinical sepsis score at 24 h post treatment of hACE2$^+$ and hACE2$^-$ animals treated with LPS, or LPS and spike protein. **f,** Quantification and representative micrographs of microthrombi in lungs of hACE2$^+$ and hACE2$^-$ animals treated with LPS, or LPS and spike protein. Vessel borders are shown with dashed white lines. Scale bar 50 µm. **g,** Quantification of fibrinogen-positive neutrophils in vessels in the lungs of hACE2$^+$ and hACE2$^-$ animals treated with LPS, or LPS and spike protein. **e-g:** Two tailed unpaired t-tests, n=3 per group. **h,** Quantification of microthrombi in lungs of control or

reparixin treated K18-hACE2 mice as shown in Fig 4h, in addition to LPS-treated C57BL/6 mice (WT). Representative micrograph of vessels of LPS-only treated WT animals shown, representative micrographs of the other groups shown in **4h**. **i,** Clinical sepsis score at 4h post S1 Spike protein-induced lung injury for control and reparixin treated hACE2 mice. **h-i:** Two tailed unpaired t-tests, n=4 per group. Mean ±sem is shown. *p<0.05, **p<0.01, ***p<0.001.

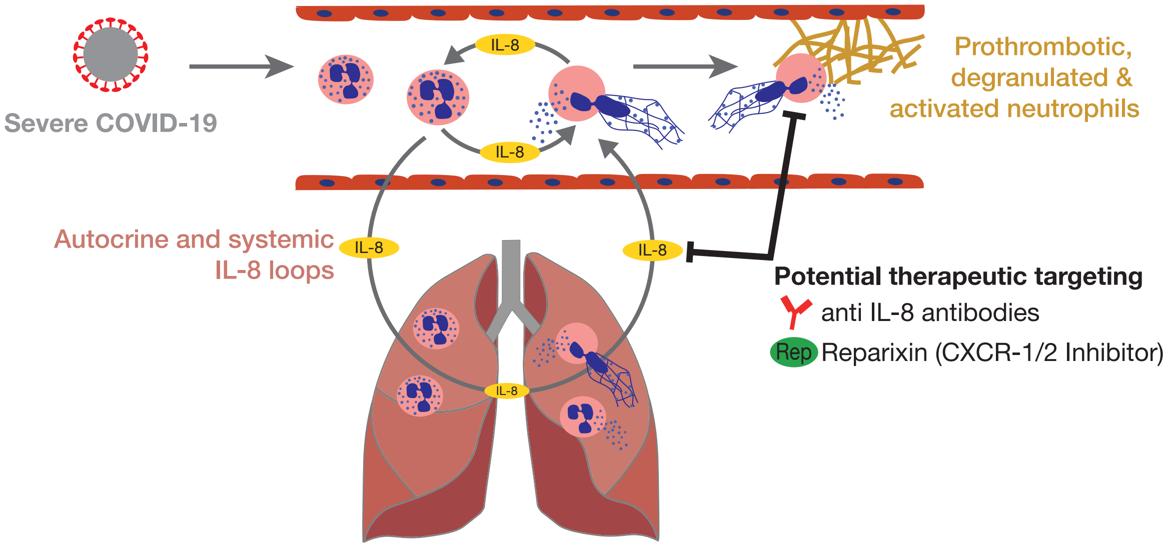

Graphical abstract. Neutrophils in the circulation and in the lung of severe COVID-19 patients are activated through systemic and autocrine interleukin 8 (IL-8) loops. These loops lead to a degranulated and prothrombotic neutrophil phenotype, especially in severe COVID-19 compared to both intermediate COVID-19 and other viral pneumonias. Therapeutic targeting of IL-8 by either anti IL-8 antibodies or the CXCR-1/-2 blocker reparixin reduces SARS-CoV-2-driven neutrophil degranulation, NETosis and pulmonary microthrombosis.

Supplemental Tables

Suppl. Table 1 | Proteome score data

ISG Score	IL-8 Score	Azurophilic Granule Score	Gelatinase Granule Score	Secretory Vesicles Score	Specific Granule Score
MX1	AIFM1	BPI	CTSD	FCGR1A	CST7
IFIT1	AKT1	LAMP1	MMP2	ALPL	HPSE
ISG15	CD74	PSEN1	TNFAIP6	CCR1	MMP2
IFIT3	CNP	VNN1	MMP8	LNPEP	SERPINA1
IFIT2	CRYZ	AZU1	TNFRSF1A	TLR8	CST3
IFIT5	CXCR1	HEXB	B2M	CD97	SLPI
XAF1	CXCR2	ARG1	GPI	CD14	TNFRSF1A
MX2	ICAM1	FUCA2	LILRA3	TLR4	CLEC5A
IFI30	IRAK4	GNS	CR1	ITM2B	CEACAM1
IFI16	ITGAL	GLB1	ARSB	TGFBR2	B2M
IFI44	LBP	ORM2	SIRPB1	AGTRAP	ITGAL
IFI35	MAPK3	CEACAM6	ATP11A	TNFRSF1A	GGH
IFITM3	MTOR	ACPP	FCER1G	IFNGR1	C3AR1
IRF9	MYD88	GALNS	FGL2	TLR1	PTX3
IRF2BP1	PLCB2	MAN2B1	VAMP8	IGF2R	VAMP2
ISG20	PSMD12	HEXA	ARG1	MYD88	STOM
IRF2BP2	PTK2B	SDCBP	CD58	TLR2	CYBA
IRF1	TLR4	CD63	FCN1	SYNGR2	SNAP23
IRF3	TLR5	ELANE	FCAR	SORL1	HP
IRF2BPL	TLR8	MPO	ITGAX	CR1	PGLYRP1
	VEGFA	GLA	MGAM	FCGR3B	TSPAN14
		CTSG	VAMP2	TNFRSF1B	CD59
		PRTN3	CYBA	IFNAR2	FOLR3
		GUSB	SCAMP2	ANTXR2	PLAU
		LYZ	TNFSF14	DYSF	PLAUR
			MMP25	TLR6	CYBB
**TCC Score**	**C1 Score**	**Fibrinogen Score**	VNN2	FCAR	ITGB2
C9	C1R	FGA	LILRB2	VAMP2	ITGAM
C8G	C1S	FGB	SIGLEC5	CYBA	TCN1
C8B	C1QA	FGG	TIMP2	MMP25	CEACAM8
C8A	C1QB		SNAP23	LILRB2	ORM1
C7	C1QC		TCIRG1	SNAP23	OLFM4
C6			PRSS3	IL6R	LYZ
C5			ADAM8	FCGRT	LTF
			MMP9	PLXNC1	CHI3L1
			ASAH1	FPR1	LCN2
			PRCP	SIGLEC9	CRISP3
			CTSB	P2RX1	CAMP
			PLAUR	PLP2	
			CD33	FCGR3A	
			SLC11A1	LILRB3	
			CYBB	CYBB	
			LAMP2	ANPEP	
			ITGB2	CXCR1	
			CTSH	ITGB2	
			ITGAM	ITGAM	
			CECR1	TREM1	
			TCN1	CXCR2	
			CEACAM8	MME	
			ORM1		
			CTSZ		
			CTSS		
			LYZ		
			CRISP3		
			CAMP		

Suppl. Table 2 | Cell type annotation Liao et al.				
**cluster**	**all**	**control**	**mild**	**severe**
Monocytic	48893	17857	4157	26879
T cells	7533	760	2333	4440
Plasma cells	1179	1	7	1171
Epithelial	2275	50	219	2006
Dendritic cells	1224	518	356	350
NK cells	1624	19	175	1430
Neutrophils	810	0	3	807
B cells memory	202	18	66	118

Suppl. Table 3 | Cell type annotation Wauters et al.					
**Cluster**	**Cell Number**	**Covid_mild**	**Covid_severe**	**Pneu_mild**	**Pneu_severe**
**AT2**	866	1	245	431	189
**Alveolar_macrophage**	1960	26	538	1391	5
**B_cell**	439	8	387	44	0
**Basal**	1110	24	579	466	41
**CD4_Tcell**	2722	38	2063	621	0
**CD8_Tcell**	1455	45	1033	376	1
**Ciliated**	6801	238	3707	2479	377
**Inflammatory**	636	3	357	276	0
**Mast_cell**	325	1	29	295	0
**Md_macrophage**	21263	792	4191	16237	43
**Monocyte**	5883	248	3554	2003	78
**NK**	790	26	592	172	0
**Neutrophil**	7337	431	6117	788	1
**Plasma_cell**	1294	5	1191	92	6
**Secretory**	3687	26	1969	999	693
**Squamous_KRT13**	3093	103	1551	1349	90
**cDC**	397	31	219	145	2
**pDC**	100	0	72	28	0
**Total**	60158	2046	28394	28192	1526

**Suppl. Table 4 | Data of patients included in OLINK CXCL8 plasma level measurements**			
**Cohort**	**Number included**	**Median Age [IQR]**	**% Female**
**Non-COVID-19 control patients**	26	73.0 [53.0,77.8]	23.1%
**Mild to Moderate COVID-19 (WHO 1-4)**	78	60.0 [47.3,75.8]	29.5%
**Severe COVID-19 (WHO 5-8)**	29	72.0 [65.0,75.0]	41.4%

**Suppl Table 5 | ClueGo Term Name (Suppl. Fig. 1f)**
antigen processing and presentation of peptide antigen via MHC class I
acute inflammatory response
production of molecular mediator involved in inflammatory response
pre-replicative complex assembly involved in nuclear cell cycle DNA replication
DNA unwinding involved in DNA replication
DNA replication initiation
macrophage cytokine production
DNA duplex unwinding
chemokine production
interleukin-8 production
regulation of interleukin-8 production
negative regulation of tumor necrosis factor production
positive regulation of chemokine production
regulation of toll-like receptor signaling pathway
negative regulation of toll-like receptor signaling pathway
positive regulation of cytokine biosynthetic process
interleukin-8 biosynthetic process
positive regulation of nitric oxide biosynthetic process
interleukin-1 secretion
interleukin-1 beta secretion
regulation of interleukin-1 secretion
interferon-gamma-mediated signaling pathway
positive regulation of pattern recognition receptor signaling pathway
cellular response to interferon-gamma
detection of external biotic stimulus
alpha-amino acid biosynthetic process
